# Highly regulated growth and development of the Ediacara macrofossil *Dickinsonia costata*

**DOI:** 10.1371/journal.pone.0176874

**Published:** 2017-05-17

**Authors:** Scott D. Evans, Mary L. Droser, James G. Gehling

**Affiliations:** 1 Department of Earth Sciences, University of California at Riverside, Riverside, California, United States of America; 2 South Australia Museum, Adelaide, South Australia, Australia; NORWAY

## Abstract

The Ediacara Biota represents the oldest fossil evidence for the appearance of animals but linking these taxa to specific clades has proved challenging. *Dickinsonia* is an abundant, apparently bilaterally symmetrical Ediacara fossil with uncertain affinities. We identified and measured key morphological features of over 900 specimens of *Dickinsonia costata* from the Ediacara Member, South Australia to characterize patterns in growth and morphology. Here we show that development in *Dickinsonia costata* was surprisingly highly regulated to maintain an ovoid shape via terminal addition and the predictable expansion of modules. This result, along with other characters found in *Dickinsonia* suggests that it does not belong within known animal groups, but that it utilized some of the developmental gene networks of bilaterians, a result predicted by gene sequencing of basal metazoans but previously unidentified in the fossil record. *Dickinsonia* thus represents an extinct clade located between sponges and the last common ancestor of Protostomes and Deuterostomes, and likely belongs within the Eumetazoa.

## Introduction

The Ediacara Biota is generally accepted as the first occurrence of macroscopic, complex, animals in the fossil record [1,2]. Predictions based on gene sequencing of basal metazoans suggest that within these early communities, in addition to ancestral animal forms, we should find extinct lineages that do not fit within known animal phyla [3]. Despite this, previous attempts to classify Ediacara fossils have focused on placing them within extant animal clades and thus have proved unsuccessful, leaving significant gaps in our understanding of early animal evolution. Recent work focused on determining relationships within the Ediacara biota based on morphological similarity has demonstrated the utility of interpreting characters of these organisms independent of previously recognized phylogenetic schemes [1,4]. *Dickinsonia* is an abundant member of the Ediacara Biota that was mobile and seemingly complex [5]. Despite numerous interpretations [6–8], from fungi [9] to annelids [10,11], and recently to placozoans [12] and bilaterians [13], the phylogenetic placement of *Dickinsonia* remains controversial [14].

Specimens of *Dickinsonia* occur in the Ediacara Member of the Rawnsley Quartzite, cropping out in the Flinders Ranges and surrounding areas of South Australia (S1 Fig). Ediacara Member fossils are preserved in sandstones characterized by episodic deposition [15,16]. Early mineralization of these deposits after burial yields exceptional preservation of organisms such as *Dickinsonia* as external molds in negative relief on the bases of beds [15,17]. The majority of specimens are in excellent condition indicative of *in situ* preservation. However, *Dickinsonia* is found in a range of preservational modes, including folded, ripped and clearly transported individuals, and some with evidence of death prior to burial [16,18]. These factors make it critical that an abundance of specimens be examined to eliminate taphonomic processes as a cause of morphologic variability.

**Fig 1:**
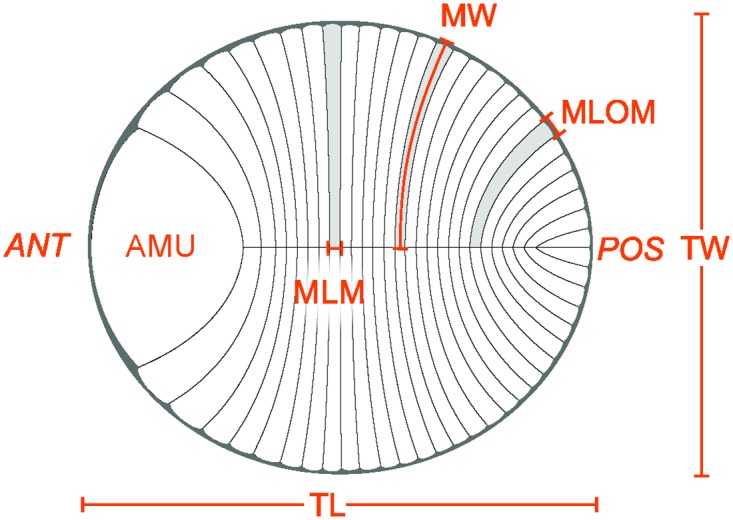
Illustration of representative *Dickinsonia costata*. Labels represent posterior (POS), anterior (ANT), total length (TL) and width (TW), module length at outer margin (MLOM) and midline (MLM), module width (MW) and anterior most unit (AMU). Illustration by Sohail Wasif.

In the Ediacara Member *Dickinsonia costata* is the most abundant of the five currently recognized species of *Dickinsonia*. Current species distinctions are based largely on overall shape and size as well as the size of modules [19–22]. Compared to other species *D*. *costata* is ovoid in shape and has the fewest number of modules per unit length. We use the terms anterior and posterior (see Fig 1 for all morphological characters) as defined by the inferred direction of movement [5]. *Dickinsonia* contains a midline running parallel to the long axis of the body and is divided into numerous repeated features that have been variously referred to as segments [5,9,10] or modules [12,18]. Segments are conservatively defined as repeated units along the anterior-posterior axis containing anterior-posterior polarity within individual units [23]. No such polarity within units has been recognized for *Dickinsonia*, so we refer to them as modules following previous authors [12,18]. Modules are smallest at the posterior end [5,11–14]. The anterior most unit is distinct from other modules in that it is not divided by the midline. All modules, as well as the anterior most unit, terminate at a smooth, well-defined outer margin. Length refers to any feature that for the majority of modules is approximately parallel to the long axis of a specimen and width is parallel to the short axis.

Previous descriptions of *Dickinsonia*, relying on relatively few specimens, have presented conflicting views on morphology [5,13,14,24–30]. Some authors have suggested that the modules of *Dickinsonia* were offset at the midline, invoking a glide plane of symmetry [24–26], others contend that modules run continuously through the midline and that the organism was bilaterally symmetrical [5,13] and some have claimed that both forms are present [14]. Further, it has recently been suggested that the modules of *Dickinsonia* bifurcated, merged and changed in relief [26]. Other reports have suggested that *Dickinsonia* possessed complex internal structures [27–30].

Here we present analyses of a significant number of specimens to refine the morphology of *D*. *costata*. In addition, we collected measurements of key morphological characters that chronicle the growth and development of this organism to determine how it fits in the early evolution of animal life. Our results indicate that *Dickinsonia* represents a previously unrecognized lineage of eumetazoans that utilized some of the gene regulatory networks found in bilaterians and likely went extinct prior to the rise of more recognizable animal forms during the Cambrian.

## Materials and methods

We photographed, documented and observed morphologic variation in 988 specimens of *D*. *costata* from the South Australia Museum (SAM) in Adelaide and Nilpena, a field site west of the Flinders Ranges, South Australia. The Nilpena site is a privately owned property and permission to conduct this research was granted via the landowners Ross and Jane Fargher (see acknowledgements). Ongoing excavation at Nilpena over the last 15 years has resulted in the exhumation of 28 beds and over 300 m^2^ of *in situ* fossiliferous material (see Joel et al [31] for further description of bed excavation). All figured specimens are either deposited at the SAM (P53893, P41202 and P41074) where they are publicly accessible, or, in the case of specimens from Nilpena (1TFB-01 and MM3-01), are preserved on *in situ* bedding planes and cannot be removed from the site.

Of the 988 total specimens examined here, length and width were measured from 538 complete specimens with no evidence of deformation using digital calipers on original specimens or latex molds. The number of modules was counted directly from fossil specimens preserved well enough to consistently identify discrete modules. This process yielded 194 specimens for which we could accurately determine module numbers. Reported module numbers necessarily represent minimum estimates as modules near the posterior end of many specimens become smaller than the resolution of the grains in which they are preserved. Simple linear regression models were conducted using the Minitab^®^ Statistical Software and p-values are reported for F- (analysis of variance or ANOVA) and t-tests.

**Fig 2:**
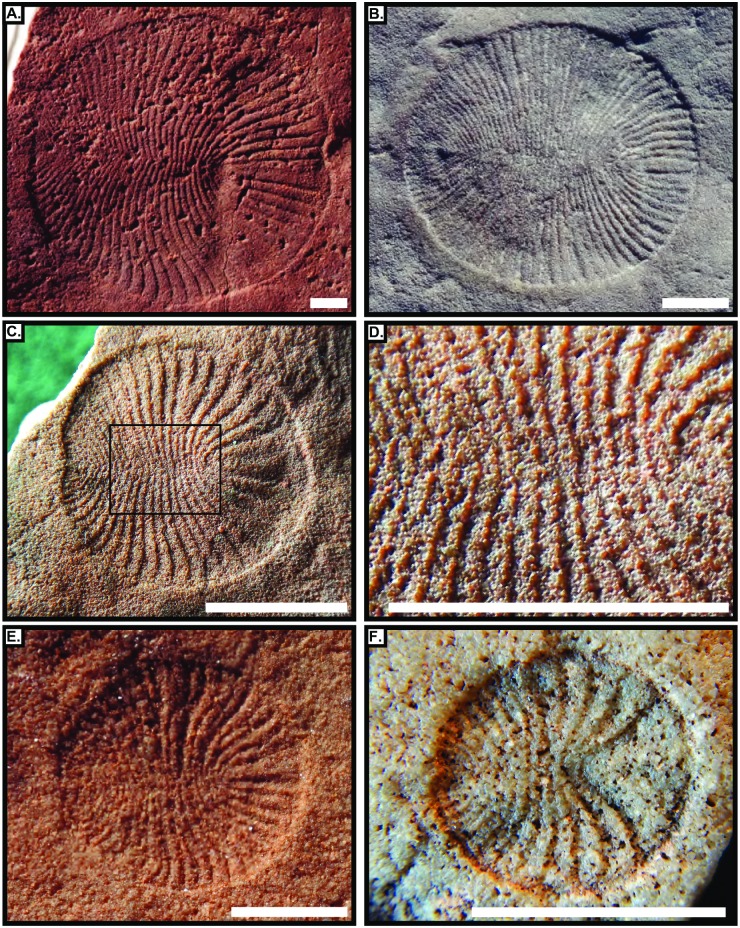
Representative fossil specimens of *D*. *costata*. Specimens from the Ediacara Member, Rawnsley Quartzite used to demonstrate growth patterns. Notice the clear bilateral symmetry and smooth, well-defined outer margin in all specimens. The box in panel C represents the zoomed in area shown in panel D. (A) SAM P53893. (B) 1TFB-01. (C,D) SAM P41202. (E) MM3-01. (F) SAM P41074. Scale bars are 1 cm.

Module length at the midline and outer edge as well as module width were measured on the 94 best preserved specimens using photographs and the Image J software available at https://imagej.nih.gov/ij/. These specimens were chosen based on the ability to measure individual module features for a significant (>75%) portion of the specimens total modules. Module lengths were measured as straight-line distances at the midline and outer edge. Module width was measured along the sinusoidal path of the module from midline to outer edge. For graphical representation we chose five exemplary samples (Fig 2) that accurately summarize the findings of this analysis (see S2 and S3 Figs for analysis of 5 additional specimens). We calculated the average increase in module length at the outer margin as the sum of the outer margin module length on the right and on the left side for each module, minus the sum of these lengths for the module located immediately posterior, divided by the sum of the module length at the outer margin for the module. Measurements of length and width were obtained from the anterior most unit and compared to the sum of the module lengths at the outer margin for the right and left side of the first anterior module.

## Results and discussion

### Morphological observations

This investigation of over 900 specimens of *D*. *costata* represents the largest dataset of this taxon analyzed to date, thus illuminating which features are representative of organismal biology and those that represent taphonomic artifacts. The shape of *D*. *costata* is consistently ovoid in all specimens investigated except in rare examples that have obviously been altered by taphonomic processes. The best-preserved specimens of *D*. *costata* clearly show that modules are continuous across the midline (Fig 2D). It is highly unlikely that this precise matching is caused by distortion, suggesting that this organism was bilaterally symmetrical. Further, close examination demonstrates that any apparent evidence for modules being offset at the midline is the result of alteration due to the soft-bodied nature of *Dickinsonia* and that modules are consistently symmetric about the long axis in all specimens. This indicates that previously reported evidence for offset modules [24–26] is likely the result of taphonomic distortion, which is probable given the variable preservation of *Dickinsonia*, or could reflect a previously unrecognized species distinction between specimens from South Australia, with bilateral symmetry, and those with reported offset symmetry from elsewhere. There is no evidence that modules bifurcate or merge in a biologically meaningful way and all modules in body fossils of *D*. *costata* are preserved in varying degrees of negative relief. While individual specimens may appear to have these features, their occurrence is rare and can be attributed to taphonomic deformation. No evidence for internal structures was observed in any specimens analyzed herein. All previous reports of features such as a through gut are likely due to deformation or the draping of *Dickinsonia* over irregular features present on the Ediacaran seafloor.

### Overall growth

**Fig 3:**
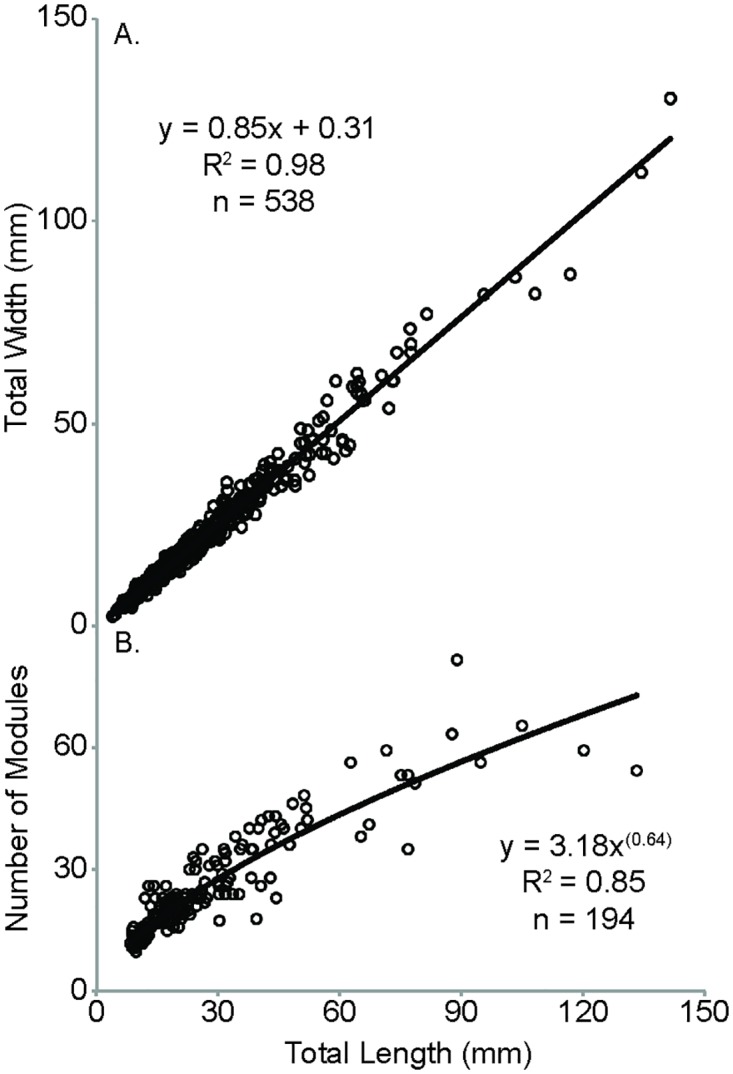
Graphical representation of overall growth. (A) Total width, and (B) number of modules versus total length of *D*. *costata* with best-fit line plotted, equation and R^2^ of best-fit as well as total number of specimens (n).

Measurements of total length for *D*. *costata* range from 4.15 to 140.55 mm with a mean value of 24.31 mm and total width ranges from 3.38 to 130.11 mm with a mean value of 21.06 mm. The relationship between overall length and width is strongly linear (R^2^ = 0.98) and through the origin (Fig 3A). Linear regression models support a statistically significant correlation between total length and width (p < 0.0001 for both F- and t-tests). Height is not easily resolved from specimens of *Dickinsonia* and fluctuations in height are not singularly controlled by biological factors due to compaction and taphonomic variability. There is no evidence to suggest that taphonomic effects are size dependent and changes in height are insignificant with respect to length and width. Typically, the preserved height of *D*. *costata* is less than 1 to 2 mm and rarely greater than 5 mm in total relief. These results are consistent with previous examinations of this species [6,9,11].

Our data are in agreement with previous reports [6,9,11] suggesting isometric growth of *D*. *costata* in terms of total length and width. This result is especially striking when we consider the soft-bodied nature of this organism. The linear trend indicates that overall length and width increased throughout the life of *D*. *costata* and that the co-variation between these metrics represents one of the strongest constraints on growth. The consistency of the length to width relationship, as well as the lack of significant variation with respect to height, indicates that maximizing the surface area to volume ratio was an important factor in the development of *D*. *costata* [11]. Maintaining a consistent length to width ratio also likely contributed to the conservation of an overall ovoid shape.

There is a moderate (R^2^ = 0.77) positive linear relationship between total length and number of modules, however a power function yields a slightly stronger trend (R^2^ = 0.85; Fig 3B). Linear regression models support a statistically significant correlation between total length and number of modules (p < 0.0001 for both F- and t-tests). In general this positive relationship indicates that as *D*. *costata* grew the number of modules increased. The slightly better fit of a power law suggests that there may be an upper limit to the number of modules in *D*. *costata* and that the organism added fewer modules the larger it became.

Despite this moderate trend in module number relative to size, some individuals have up to three times as many modules as those with similar overall lengths. The inverse relation is also identified in specimens where length can be more than three times greater in one specimen than in another with a similar number of modules. While these examples represent extreme end members of the overall distribution, it is common to find specimens with the same number of modules that vary in size by at least a factor of two. The variability in module number versus total length cannot be attributed to currently recognized species distinctions and the continuum of values in Fig 3B suggests that the plasticity of module numbers is not due to the presence of multiple unrecognized species. The inconsistency of module number with respect to size and the limited number of relatively large specimens prevent any definitive conclusions but suggest that module number and body size are only slightly correlated. Runnegar [6] attributed this difference to the expansion and contraction of *Dickinsonia*. Observation of numerous variations within the general ovoid shape of this organism indicates that *D*. *costata* was likely capable of expansion and contraction. However, the three fold difference of module number in specimens with similar lengths and in total length in specimens with similar module numbers cannot be singularly explained by expansion and contraction, especially given the tightly constrained length to width ratio for this organism. End members of each example also do not consistently show evidence of expansion or contraction. We therefore conclude that the number of modules is not solely determined by overall size and that similarly sized specimens can have vastly different module numbers. The reasons for large differences in module number between specimens may simply be random as has been observed in the modern polychaete *Platynereis dumerilii* in which siblings living in close association have been observed with vastly different numbers of segments [32]. This suggests that there was likely little functional significance in maintaining a specific number of modules with respect to size and highlights that conserving an ovoid shape and consistent length to width ratio was critical for *D*. *costata*.

### Growth of modules

**Fig 4:**
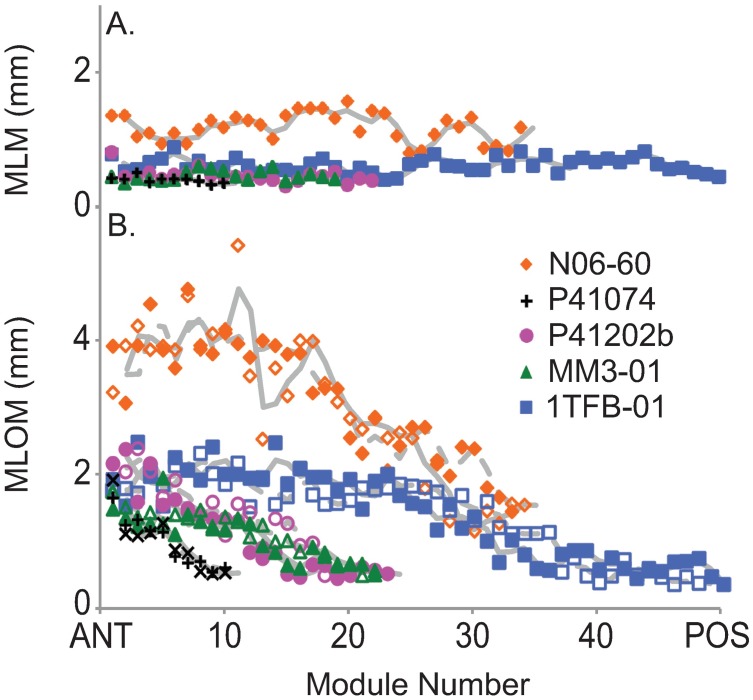
Graphical representation of changes in module length. (A) Module lengths along the midline (MLM), and (B) module lengths along the outer margin (MLOM) versus number of modules for the five specimens shown in Fig 2 of *D*. *costata*. Moving from anterior (ANT) to posterior (POS) from left to right on the x-axis. Grey trend lines represent two point moving averages. Open and closed shapes in (B) represent opposite sides of the same specimen, dotted trend lines correspond to open shapes.

Comparison of module lengths at the midline and outer edge of *D*. *costata* demonstrates two distinct trends (Fig 4). Module lengths along the midline do not vary within an individual except at the anterior-most end where the first few modules are rarely much larger than subsequent modules (S2A Fig). At the posterior, where new modules are added, module lengths similarly do not vary at the midline. There is a weak (R^2^ = 0.64) positive correlation between average module lengths along the midline and total length indicating that at the midline, all module lengths increased uniformly with growth (S4 Fig).

The consistency along the midline of *D*. *costata* suggests that module length increased rapidly when initially inserted to match previous modules at the midline. Once the length of an inserted module reached that of preceding modules at the midline, it grew at the same rate as all other modules, getting larger along with total length. The conservation of midline length is noteworthy given the irregularity of module numbers relative to overall size and suggests that, like total length and width, maintaining module length along the midline was a constraining factor in the growth of *D*. *costata*. The consistency of module lengths within individual specimens also indicates that modules were in some way fixed at the midline.

Module lengths at the outer margin decrease from anterior to posterior regardless of specimen size (Fig 4B). This suggests that the length of individual modules at the outer margin expanded consistently through life. Typically module lengths at the outer margin increase with total length linearly. The average increase in module length from adjacent modules at the outer margin is 4.01 ± 13.23% (SD). Measurements of the length at the outer margin for the anterior most unit show that in 29 out of the 94 best preserved specimens this feature is more than 20% larger, and in 13 specimens more than 50% larger than the sum of module length at the outer margin for the right and left side of the first true anterior module (e.g. Fig 2F).

Previous reports have suggested that *Dickinsonia* grew by the posterior addition of modules, based on the observation that modules are smallest at the posterior end [13]. The lack of any branching modules or smaller intercalated modules in the hundreds of specimens analyzed indicates that they are not added between the posterior and anterior end by bifurcation of pre-existing modules. It is reasonable then to conclude that modules must either be added at the posterior or anterior end. Grain size limitation does not allow detailed examination of the posterior-most modules. However, the lack of any branching in the anterior-most module, which is large enough to be clearly seen in most specimens, suggests that modules are not released at the anterior end. The presence of specimens with the anterior most unit significantly larger than the proceeding modules provides additional evidence that modules were not added at the anterior end. Thus our data demonstrate that the most parsimonious explanation for module addition is that they were added at the posterior end.

**Fig 5:**
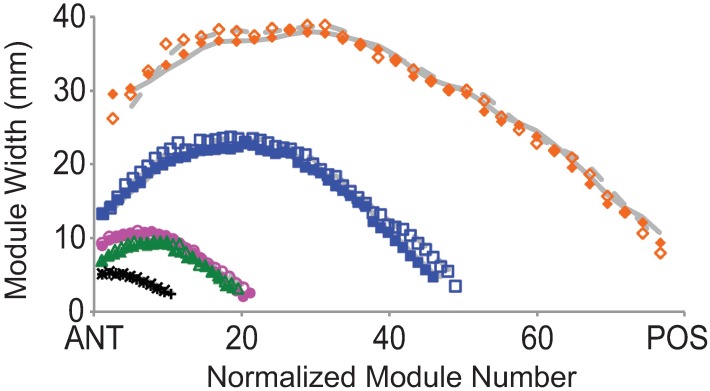
Graphical representation of changes in module width. Module width (MW) versus normalized module number for *D*. *costata*. Moving from anterior (ANT) to posterior (POS) from left to right on the x-axis for the five specimens shown in Figs 2 and 4 of *D*. *costata*. Grey trend lines represent two point moving averages. Shapes and colors represent the same specimens from Fig 4. Dotted trend lines correspond to open shapes. Module number is normalized to total length by dividing the module number by the total number of modules and multiplying by total length. Open and closed shapes represent opposite sides of the same specimen.

*Dickinsonia costata* module widths are smallest at the posterior end, increase up to roughly the middle of the long axis and then decrease towards the anterior end (Fig 5). Within specimens anterior-most modules are still larger than posterior-most modules. This indicates that while module widths increased through growth, a given module increased faster when it was at the posterior half of the organism. This trend is observed consistently in all specimens regardless of total length or module number.

**Fig 6:**
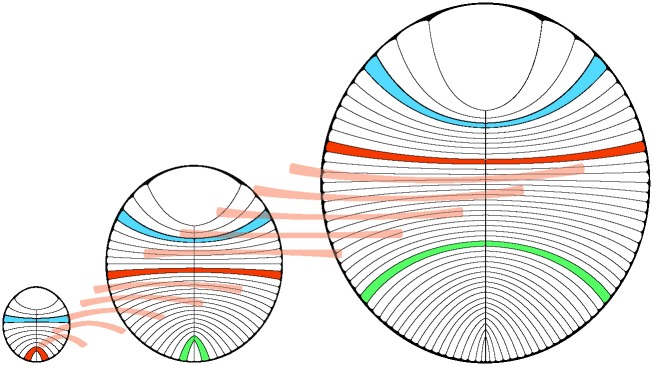
Idealized cartoon of *D*. *costata* growth. Illustration demonstrating the changes that occur in module shape and size with increases in total length and width. Illustration by Sohail Wasif.

The different growth rates for the characters discussed above result in variable module shapes both between specimens and within individual specimens of *D*. *costata* (Fig 6). In general though, posterior modules run straight from the midline to the outer edge at some angle less than 90 degrees forming a roughly “v” shape. From the posterior towards the middle of the long axis of a specimen this angle increases and becomes perpendicular to the midline. From this point to the anterior of a specimen modules typically bend so that they are still approximately perpendicular where they intersect the midline but become roughly parallel to the midline closer to the outer edge, forming “u” shaped modules. The soft-bodied nature of *D*. *costata* leads to many variations preserved within this approximate trend in module shape change. The “bending” of anterior modules indicates that modules must have been fixed not only at the midline, but also at the outer margin to some type of membrane. Increases in overall module size must have occurred in concert with the growth of this membrane. The variations in module width and length at the outer margin from one side to another within even the most pristine specimens further suggest that this outer membrane must have been somewhat flexible, but rigid enough to regulate predictable changes in module shape.

The observed variations in module size, growth rate and shape appear to reflect the importance of maintaining the characteristic ovoid shape of *D*. *costata* while accommodating growth. Modules grew longer at the outer margin and wider to increase the size of the organism. Variable growth patterns of module widths and changes in module shape were adjusted during growth to maintain an ovoid morphology (similar to the parabolic description of segments by Runnegar [11]). This constrained growth pattern likely occurred in association with, or as a consequence of a tough yet pliable outer membrane. Our data demonstrate that despite an apparently simplistic morphology, *D*. *costata* modules grew by terminal addition and module inflation in a highly regulated and complex manner.

### Comparison with other taxa

Other Ediacaran organisms, such as *Charnia*, grew by the addition and inflation of modular body divisions to achieve relatively large sizes [33]. These Rangeomorph taxa grew by the repetition and branching of self-similar units, creating the characteristic fractal body pattern and achieving maximum surface area by 3D space filling [34]. In contrast, *D*. *costata* grew to maximize 2D space, surface area to volume ratios, and all aspects of module inflation were regulated to maintain an ovoid shape. This suggests that rather than close phylogenetic relation between these two groups, that the addition and inflation of modular units was a common growth strategy and that the underlying regulatory genes that produce this style of growth may be present in a diversity of disparate forms within the Ediacara Biota. This result also demonstrates that there were diverse growth strategies in which modular Ediacaran organisms could maximize surface area, particularly while attaining large body size.

A natural comparison arises between the growth of segmented animals (annelids, arthropods and vertebrates) and *D*. *costata*. There are many obvious differences between these groups beyond the definition of a true segment discussed above, for instance all truly segmented animals have a trunk composed of segments that is distinct from the head and tail [23]. Further, while there is a wide range of growth patterns found in the diverse array of known segmented organisms, those patterns typically follow specific rules. For example, arthropods grow by molting, and many arthropods have a constant growth rate per-molt, the so-called Dyar’s rule [35]. Because segment addition occurs in association with molting, size and number of segments, as well as the number of molts and thus age, are strongly correlated [36,37]. The plasticity of module number with respect to overall size between specimens of *D*. *costata*, despite the tight regulation on modular growth, suggests that module number is not a reliable proxy for age and that different specimens add and inflate modules at variable rates. This suggests that growth in *Dickinsonia* is fundamentally different from that of truly segmented animals.

We are not currently aware of any modern or extinct organism, segmented or otherwise, that grows in the same manner as *D*. *costata*. Any convergence between the growth of *D*. *costata* and modern organisms would likely reflect the importance of maintaining an ovoid shape and not phylogenetic ancestry. This is consistent with previous explanations for the morphological similarities between *D*. *costata* and modern organism that are most likely unrelated [11,13].

*Dickinsonia* was one of the few mobile Ediacara taxa [5] and it possibly fed via external digestion of organic matter through its ventral surface, leading to the hypothesis that it may have been related to modern Placozoa [12]. In terms of growth, *Trichoplax adhaerens*, the only known species of placozoan, is highly irregular, with increasing variability as size increases, and has even been reported to change from circular to elongate in successive generations of asexually reproducing populations [38–41]. Individuals can also change dramatically in terms of both shape and size without truly growing [38]. These large fluctuations are inconsistent with the tight constraint on overall body shape observed for *D*. *costata*. This result does not exclude a placozoan affinity for *D*. *costata*, but it highlights a major difference in growth between the two organisms. The large discrepancy in growth patterns between *Dickinsonia* and placozoans indicates that the overlapping characters between the two groups are more likely due to similarities in function rather than reflective of phylogenetic ancestry.

### Phylogenetic placement

It is generally agreed that the split between bilaterians and other animals occurred prior to the evolution of *Dickinsonia* [42]. Fossils of bilaterians have been identified from the Ediacara biota; the furrowed trace fossil *Helminthoidichnites* is widely accepted as evidence of bilaterians and the body fossil *Kimberella* is largely accepted as a bilaterian and has been reconstructed as a stem group mollusk [3, 42,43] but see [14] for discussion. The highly regulated growth of *Dickinsonia*, along with features such as posterior addition, bilateral symmetry and organization around an anterior-posterior axis are characteristics found in bilaterians. However, most bilaterians are triploblastic and have a through gut and there is no evidence for the number of tissue layers or the presence of a mouth, anus or any type of gut in *Dickinsonia*. Some highly derived modern bilaterians do not have a through gut [44] and many studies have demonstrated the importance of the secondary loss of characters in phylogenetic reconstructions [45], but it is unlikely that *Dickinsonia* is highly derived and our results suggest that it does not have the suite of characters necessary to be considered a crown group bilaterian. The latest attempt to classify *Dickinsonia* allied it with bilaterians, either as part of the stem or crown group, based on the likelihood that growth by terminal addition did not extend beyond ancestral bilaterians in the animal tree [13]. Recent phylogenetic analysis suggests that the ancestral bilaterian was an unsegmented, benthic, ciliated, acoelomate that was likely meiofaunal and contained a “blind-gut” [46–48]. *Dickinsonia* was very sturdy and our analysis suggests that it had an outer membrane, but there is no evidence as to the total number of tissue layers. It reached relatively large sizes, with *D*. *rex* known to be as large as 1 meter in total length. It was mobile, bilaterally symmetrical, and likely obtained nutrients though external ventral digestion [5,12]. It is modular but it is possible that this is not a precursor to, or otherwise homologous with, segmentation in bilaterian clades. The presence of features likely characteristic of more derived bilaterians, such as large body size, and lack of those thought to be present in the ancestral bilaterian, such as any type of gut, make the placement of *Dickinsonia* within the stem group unlikely. However, these characters are reliant on problematic ancestral state reconstructions [49,50] so the possibility of *Dickinsonia* as a precursor to bilaterians cannot be ruled out.

The discovery of developmental patterns in *D*. *costata* that were used to conserve an ovoid shape demonstrates that growth was complex and surprisingly well regulated. The unique set of features exhibited by *D*. *costata* supports the hypothesis proposed by Erwin and Davidson [51] and corroborated by gene sequencing of basal metazoan [3] that the gene regulatory networks needed to produce the complex morphologies of bilaterians were present in more ancestral animals. In this hypothesis *Dickinsonia* would represent part of an extinct lineage that split somewhere between sponges and the LCA of Protostomes and Deuterostomes and took advantage of particular developmental gene networks common to cnidarians and higher-grade animals, but not all of those found in modern bilaterians [3,51,52]. The relative complexity of growth along with the identification of an outer tissue layer, when considered with all other features of *D*. *costata*, further suggests that this lineage likely belongs within the Eumetazoa. Recent analysis has shown that microRNAs evolved independently multiple times, suggesting that convergence cannot be ruled out when considering relations based on morphological similarities [53]. While the available data is not sufficient to eliminate convergent evolution as a possible explanation for these shared characters, the number of similar traits that are related to the gene regulatory networks found in all animals suggest that the most parsimonious placement for *Dickinsonia* is as an extinct lineage of Eumetazoa.

Traditionally, taxa of the Ediacara Biota have either been shoehorned into modern clades [6,9,10,12] or, in complete contrast considered as a group, an extinct phylum unrelated to animals [7,8]. It has recently been suggested that there are multiple, diverse groups within this biota, with varying potential relationships with modern taxa [1,4]. Given the current understanding of early animal evolution, it is likely that some taxa of the Ediacara Biota represent extinct lineages that belong along the animal tree, including those with bilaterian characters [3,51,52]. This study documenting for the first time, highly regulated growth of an Ediacara taxon, suggests that *Dickinsonia* may represent one of these predicted lineages and that similar examinations of other Ediacara taxa are necessary to gain further insight into the evolutionary history of early animals.

## Supporting information

S1 Fig Map and stratigraphy of the Ediacara Member. Map of the Flinders Ranges showing outcrops of the Ediacara Member in grey and the Nilpena field locality marked with the black star, with inset of the stratigraphic section showing the position of the Ediacara Member in bold (edited from Gehling and Droser, 2009).
(PDF)

S2 Fig Module length. Graphical representation of (a) module lengths along the midline (MLM), and (b) module lengths along the outer margin (MLOM) versus number of modules for five illustrative specimens of *D*. *costata*. Moving from anterior (ANT) to posterior (POS) from left to right along the x-axis. Open and closed shapes in (b) represent opposite sides of the same specimen.
(PDF)

S3 Fig Module width. Module width (MW) versus normalized module number for *D*. *costata*. Moving from anterior (ANT) to posterior (POS) from left to right along the x-axis. Shapes and colors represent the same specimens from S2 Fig. Module number is normalized to total length by dividing the module number by the total number of modules and multiplying by total length. Open and closed shapes represent opposite sides of the same specimen.
(PDF)

S4 Fig Average module length at the midline. Graph demonstrating increase of the average module length at midline (MLM) as total length increases.
(PDF)

S1 Dataset Measurements of *Dickinsonia costata* morphological characters. (XLSX)
