# Supplementary figures and images for: Highly regulated growth and development of the Ediacara macrofossil Dickinsonia costata
Source: PLoS One. 2017 May 17;12(5):e0176874. doi: 10.1371/journal.pone.0176874 (PMC5435172; doi:10.1371/journal.pone.0176874)

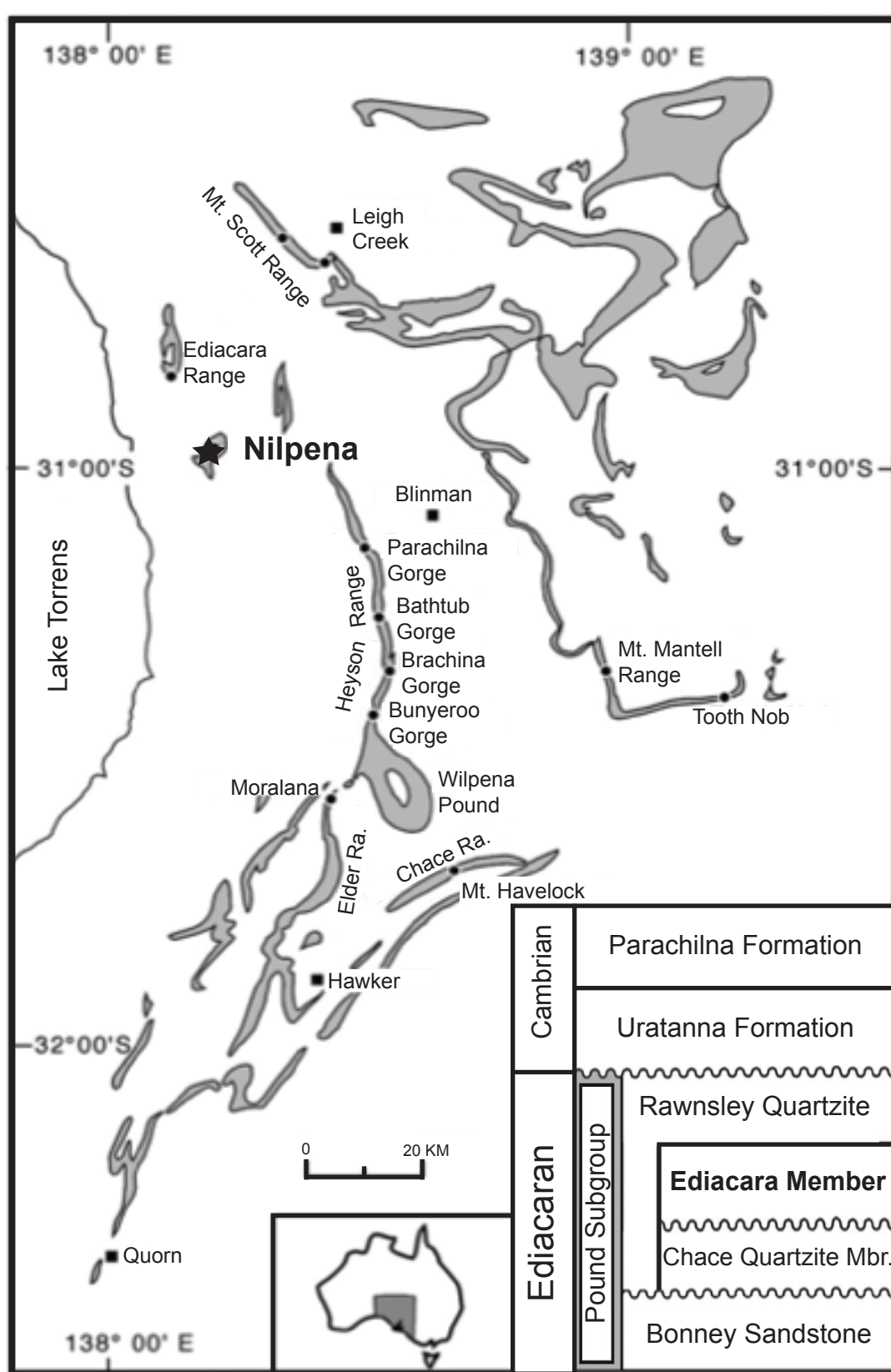

S1 Fig. Evans et al., 2017

Supplement: S1 Fig — Map of the Flinders Ranges showing outcrops of the Ediacara Member in grey and the Nilpena field locality marked with the black star, with inset of the stratigraphic section showing the position of the Ediacara Member in bold (edited from Gehling and Droser, 2009). (PDF) [file pone.0176874.s001.pdf]

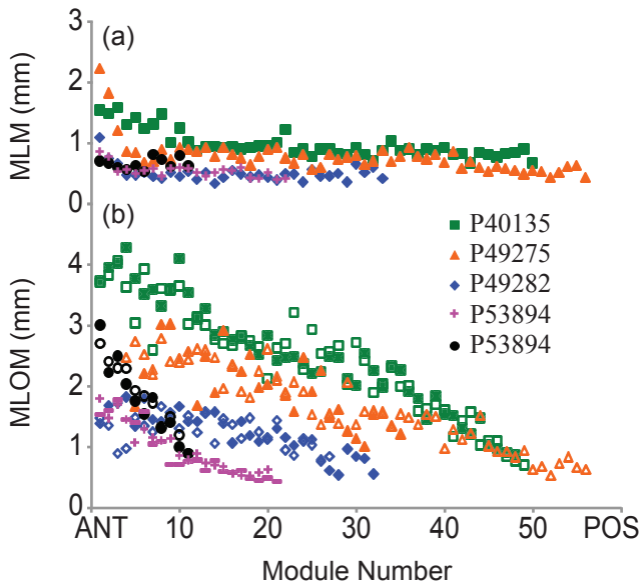

S2. Fig. Evans et al., 2017

Supplement: S2 Fig — Graphical representation of (a) module lengths along the midline (MLM), and (b) module lengths along the outer margin (MLOM) versus number of modules for five illustrative specimens of D. costata. Moving from anterior (ANT) to posterior (POS) from left to right along the x-axis. Open and closed shapes in (b) represent opposite sides of the same specimen. (PDF) [file pone.0176874.s002.pdf]

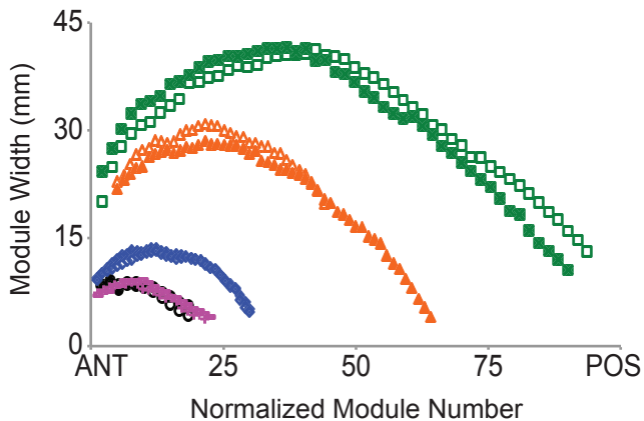

S3 Fig. Evans et al., 2017

Supplement: S3 Fig — Module width (MW) versus normalized module number for D. costata. Moving from anterior (ANT) to posterior (POS) from left to right along the x-axis. Shapes and colors represent the same specimens from S2 Fig. Module number is normalized to total length by dividing the module number by the total number of modules and multiplying by total length. Open and closed shapes represent opposite sides of the same specimen. (PDF) [file pone.0176874.s003.pdf]

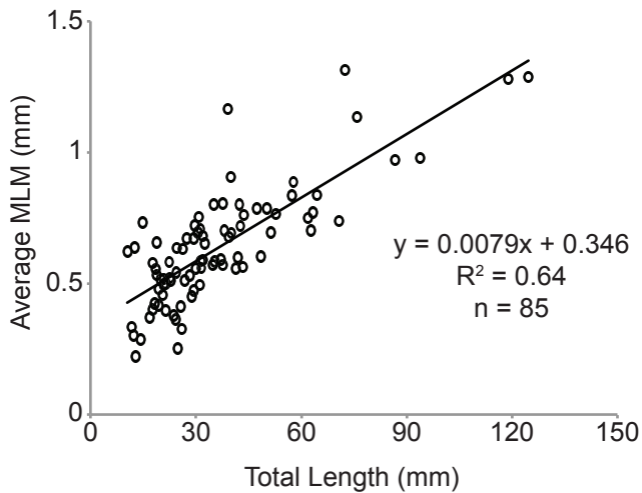

S4 Fig. Evans et al., 2017

Supplement: S4 Fig — Graph demonstrating increase of the average module length at midline (MLM) as total length increases. (PDF) [file pone.0176874.s004.pdf]
